# Do Patients with Benign Paroxysmal Positional Vertigo Have a Higher Prevalence of Osteoporosis? A Systematic Review and Meta-Analysis

**DOI:** 10.3390/jpm14030303

**Published:** 2024-03-13

**Authors:** Chul-Ho Kim, Keunho Kim, Yeonjoo Choi

**Affiliations:** 1 Department of Orthopaedic Surgery, Asan Medical Center, University of Ulsan College of Medicine, Seoul 05505, Republic of Korea; oschulhokim@amc.seoul.kr (C.-H.K.); d200626@amc.seoul.kr (K.K.); 2 Department of Otorhinolaryngology-Head and Neck Surgery, Hallym University Kangnam Sacred Heart Hospital, Hallym University College of Medicine, Seoul 07442, Republic of Korea

**Keywords:** benign paroxysmal positional vertigo, prevalence, osteoporosis, meta-analysis

## Abstract

Benign paroxysmal positional vertigo (BPPV) is a common vestibular disorder characterized by episodic vertigo. BPPV primarily affects older adults. Thus, understanding the potential relationship between BPPV and osteoporosis is clinically important. We performed a systematic search of MEDLINE (PubMed), Embase, and Cochrane Library databases for studies on the risk of osteoporosis between BPPV (+) and BPPV (−) groups up until 17 April 2023. We compared osteoporosis prevalence between groups and performed subgroup analyses for male, female, and older patients (aged ≥ 55 years). The 12 studies included 32,460 patients with BPPV and 476,304 controls. Pooled analysis showed that the BPPV (+) group had a significantly higher osteoporosis risk than the control group (odds ratio [OR], 1.73; 95% confidence interval [CI], 1.45–2.06; *p* < 0.01). Subgroup analyses also presented similar trends as male (OR, 2.41; 95% CI, 1.18–4.90; *p* = 0.02), female (OR, 2.14; 95% CI, 1.57–2.92; *p* < 0.001), and older patient subgroups (OR, 1.91; 95% CI, 1.47–2.49; *p* < 0.01) showed a higher osteoporosis risk in the BPPV (+) group than in the control group. This meta-analysis supports the hypothesis that patients with BPPV have a higher osteoporosis prevalence than those without.

## 1. Introduction

Benign paroxysmal positional vertigo (BPPV) is a common vestibular disorder characterized by episodic vertigo. Although the disease can occur in all age groups, it is more prevalent in older adults [1]. BPPV occurs when tiny particles called otoconia (also known as otoliths or canaliths) become loose and settle in the inner ear’s fluid-filled semicircular canals. There is no sensation when the head is still. However, otoconia shift within the fluid when the head moves, creating an imbalance that incorrectly triggers feelings of dizziness [2,3]. Therefore, patients with BPPV experience whirling-type vertigo when they tilt their head in a certain direction.

Although the exact etiology of BPPV remains unknown, various factors have been proposed, including head trauma, viral infections, and inner ear diseases [3]. Considering that otoliths are mainly composed of calcium carbonate, osteoporosis has been suggested as one of the etiologies of BPPV [4]. Recently, the association between BPPV and osteoporosis has attracted attention because several studies have suggested a potential link between these conditions [5,6,7]. However, evidence supporting this association remains inconclusive.

Osteoporosis is a systemic skeletal disorder characterized by decreased bone mass and microarchitectural deterioration, which can increase fracture risk [8]. It predominantly affects postmenopausal women and older adults; however, it can also occur in men and younger individuals with certain risk factors [9]. Considering that BPPV primarily affects older adults, understanding the potential relationship between BPPV and osteoporosis is clinically important.

Previous studies on the association between BPPV and osteoporosis have produced conflicting results [10]. Some studies have reported that patients with BPPV have a higher prevalence of osteoporosis, suggesting a possible shared pathophysiology or a common risk factor. Conversely, others have failed to demonstrate a significant association between BPPV and osteoporosis. To the best of our knowledge, no meta-analysis on this topic has been conducted. Therefore, the present study aimed to assess osteoporosis risk in patients with BPPV by conducting a meta-analysis. We hypothesized that patients with BPPV have a higher prevalence of osteoporosis than those without.

## 2. Materials and Methods

This study was conducted following the Revised Assessment of Multiple Systematic Reviews and Preferred Reporting Items for Systematic Reviews and Meta-Analyses guidelines [11,12]. Although this study involved human participants, neither ethical approval nor the acquisition of informed consent from participants was required because all data that were obtained were based on previously published studies and anonymously analyzed without any potential harm to the participants.

### 2.1. Literature Search

Studies exploring the correlation between BPPV and osteoporosis were searched across the MEDLINE (PubMed), Embase, and Cochrane Library databases. We identified articles published up until 17 April 2023, using a predetermined search strategy. This strategy incorporated synonyms and terms associated with both BPPV and osteoporosis. The complete search strategies and results for all databases are detailed in Supplement S1. Only studies published in English were included, with no limitations on the publication year. Following the initial electronic search, we conducted a manual search for pertinent articles and their references.

### 2.2. Study Selection

An orthopedic surgeon certified by the board and specialized in osteoporosis, along with an otologist serving as a faculty member at an academic medical center, independently chose articles for full-text review based on the titles and abstracts of the compiled studies. In cases where abstracts lacked sufficient data for decision making, the complete articles were reviewed.

The design of this meta-analysis was structured as a pairwise meta-analysis. The inclusion of studies was determined by criteria encompassing “Patient/Intervention/Comparator/Outcome/Study” criteria [13]: (1) the “population” was defined as the entire population without restrictions, (2) the “intervention” was BPPV, (3) the “comparator” was the group without BPPV, and (4) the “outcome” was the presence of osteoporosis. We only included studies on adult patients and excluded studies based on the following exclusion criteria: (1) nonoriginal articles, (2) studies that were irrelevant to the research question, and (3) duplicate studies from the same investigation group. We selected the publication with the largest population for the meta-analysis in cases where study populations overlapped.

At each stage of the article selection process, the κ-value was computed to assess inter-reviewer agreement in study selection. The agreement between reviewers was interpreted a priori using κ-values as follows: κ = 1 denoted “perfect” agreement, 1.0 > κ ≥ 0.8 indicated “almost perfect” agreement, 0.8 > κ ≥ 0.6 indicated “substantial” agreement, 0.6 > κ ≥ 0.4 indicated “moderate” agreement, 0.4 > κ ≥ 0.2 indicated “fair” agreement, and κ < 0.2 indicated “slight” agreement. Disagreements at each stage were resolved through discussion between the two investigators to achieve consensus or with the involvement of a third investigator, a board-certified otolaryngologist, in cases where consensus could not be reached.

### 2.3. Data Extraction

The following information and variables were extracted using a standardized form for qualitative data synthesis: (1) study design, (2) country in which the investigation was conducted, (3) number of patients in the BPPV (+) and control groups, (4) characteristics of control group, (5) patient age, (6) female sex, (7) methods used for diagnosing BPPV, (8) type of BPPV that was investigated, and (9) bone mineral density (BMD) T-score.

We only conducted a meta-analysis of variables for which data from three or more studies could be extracted. Therefore, we could only compare the presence of osteoporosis between the BPPV (+) and BPPV (−) groups. The T-scores of continuous variables could not be compared because of the lack of relevant study data. Furthermore, we conducted subgroup analyses to compare osteoporosis prevalence in the following subgroups: (1) males, (2) females, and (3) older adults (≥55 years of age).

### 2.4. Risk of Bias Assessment

The methodological quality of the included studies was evaluated utilizing the Methodological Index for Nonrandomized Studies (MINORS) [14], a validated tool specifically designed for assessing the quality of nonrandomized studies. The MINORS checklist comprises methodological items for nonrandomized studies (16 points) and additional criteria for comparative studies (8 points). The maximum MINORS checklist score for comparative studies is 24 points. Quality assessments were performed by two independent reviewers, and disagreements were resolved through discussion.

### 2.5. Data Synthesis and Statistical Analyses

The prevalence of osteoporosis between BPPV (+) patients and controls was the primary outcome of this meta-analysis. In the subgroup analyses, we analyzed osteoporosis prevalence in the following subgroups: (1) male, (2) female, and (3) older patients (aged ≥ 55 years).

For all comparisons, dichotomous data were analyzed, and the odds ratios (ORs) and 95% confidence intervals (CIs) were calculated. We assessed heterogeneity using the I^2^ statistic and considered 25%, 50%, and 75% as low, moderate, and high heterogeneity, respectively. Forest plots were used to present the outcomes, pooled estimates of effects, and overall summary effects of each study. Statistical significance was considered at *p* < 0.05. As previously recommended for studies in the medical field, we pooled all data using a random-effects model to avoid overestimating the study results [15]. The fixed-effects model operates under the assumption that the true effect size remains consistent across all included studies. Consequently, we considered the random-effects model to be a more generally appropriate choice for the present study. We tested for publication bias for the main outcome. However, we did not test for bias in the subgroup analyses because evaluations for publication bias are recommended only when at least 10 studies are included in a meta-analysis [16]. We conducted statistical analyses utilizing Review Manager (RevMan) software (version 5.3; The Nordic Cochrane Centre: Copenhagen, Denmark; The Cochrane Collaboration, 2014), along with the “Metafor” package in R (version 4.3.3; R Foundation for Statistical Computing, Vienna, Austria).

## 3. Results

### 3.1. Article Identification

**Figure 1:**
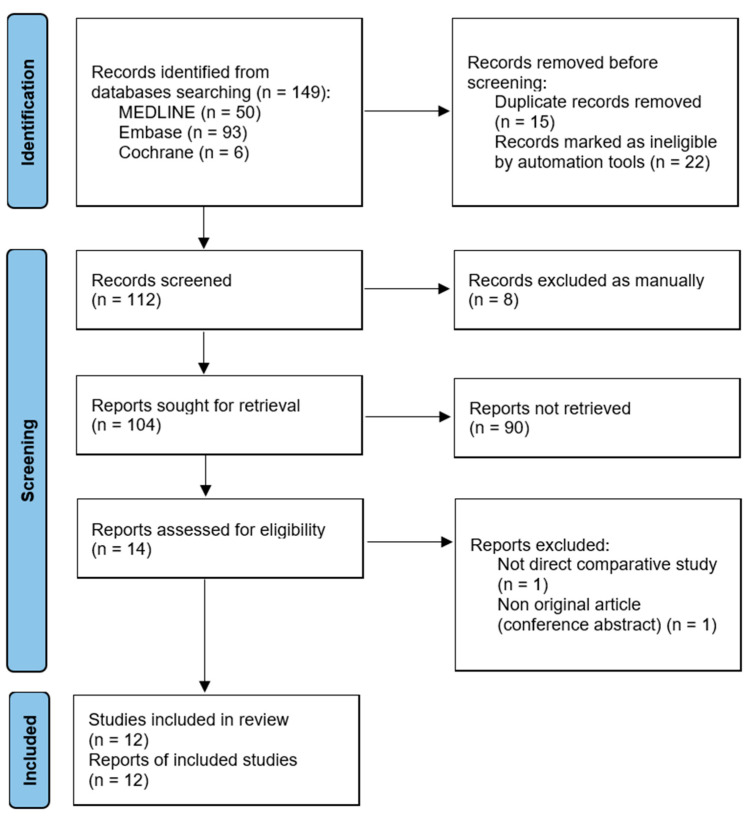
Flow diagram illustrating the identification and selection processes of studies included in the meta-analysis.

The present study’s identification and selection processes are outlined in Figure 1. Initially, a total of 149 articles were retrieved through the electronic literature search. Following the removal of 47 duplicates, 102 articles were retained for further review. Two additional articles were found through manual searching. Therefore, 104 articles were screened. Among them, 90 articles were excluded after screening their titles/abstracts, and two articles were excluded after a full-text review. Hence, 12 studies [4,6,7,17,18,19,20,21,22,23,24,25] met the criteria for both qualitative and quantitative data synthesis. The κ-values between the two reviewers demonstrated substantial agreement during the title review stage (κ = 0.730), nearly perfect agreement during the abstract review stage (κ = 0.891), and perfect agreement during the full-text review stage (κ = 1.000).

### 3.2. Study Characteristics and Qualitative Synthesis

Three studies [6,17,22] were prospective studies, and the other nine studies [4,7,18,19,20,21,23,24,25] were retrospective cohort studies. Furthermore, 10 of the 12 studies [4,6,18,19,20,21,22,23,24,25] were conducted in Asia. For the remaining studies, one was conducted in [7] North America, whereas the other [17] was conducted in South America.

The studies analyzed a total of 508,764 participants, including 32,460 patients with BPPV and 476,304 controls. With regard to participants’ mean age, the participants in most studies were in their 50s or 60s. With the exception of one study that had a male-dominant study population [23], all other studies had female-dominant study populations. The criteria used for BPPV diagnosis in each study are detailed in Table 1. Only two studies [6,24] provided details regarding BPPV subtypes. Except for two studies [7,21] that did not clearly specify their methods, all other studies appropriately diagnosed osteoporosis on the basis of BMD–dual-energy X-ray absorptiometry test results according to the World Health Organization criteria [26]. Five studies [6,22,23,24,25] described the BMD T-score in their study. However, we could not perform a meta-analysis for the T-score because of data inconsistencies. Additional details and outcomes investigated in each study are shown in Table 1.

### 3.3. Risk of Bias Assessment

The mean MINORS score for the methodological quality assessment was 18.3/24 (range: 18–20). With regard to the eight main evaluation parameters, all included studies [4,6,7,17,18,19,20,21,22,23,24,25] clearly addressed the aim of the analysis (item 1: a clearly stated aim) and appropriately included consecutive patients (item 2: inclusion of consecutive patients). Nine of the twelve studies [4,7,18,19,20,21,23,24,25] received a point deduction for their retrospective design (item 3: prospective collection of data). All included studies [4,6,7,17,18,19,20,21,22,23,24,25] appropriately stated the aim of their study (item 4: endpoints appropriate to the aim of the study). Moreover, all included studies [4,6,7,17,18,19,20,21,22,23,24,25] did not perform a blind evaluation of the objective endpoint (item 5: unbiased assessment of the study endpoint). However, the follow-up period was appropriate (item 6: follow-up period appropriate for the aim of the study). In two studies [17,22], over 5% of patients that were initially included were lost to follow-up. Each of the included studies incurred a deduction in points due to the absence of a prospectively calculated sample size (item 8: prospective calculation of study size). However, no deductions were applied to the additional criteria domains—including adequate control group, contemporary groups, baseline equivalence of groups, and adequate statistical analyses—across all included studies.

### 3.4. Meta-Analysis

#### 3.4.1. Prevalence of Osteoporosis between BPPV (+) and BPPV (−) Groups: Overall Population

**Figure 2:**
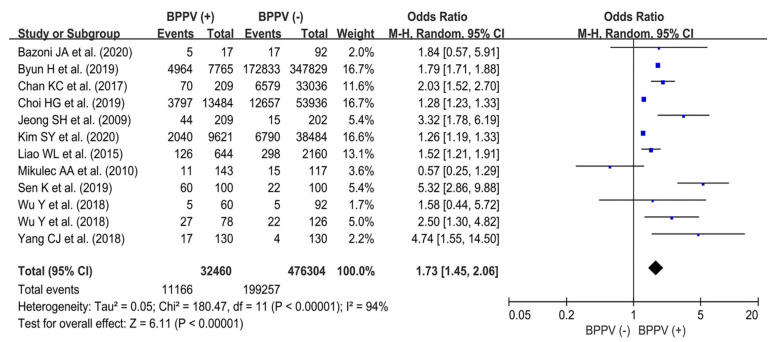
Forest plot showing osteoporosis prevalence between the BPPV (+) and BPPV (−) groups in the overall population [4,6,7,17,18,19,20,21,22,23,24,25].

The 12 included studies [4,6,7,17,18,19,20,21,22,23,24,25] investigated osteoporosis prevalence between BPPV (+) and BPPV (−) groups. A total of 11,166/32,460 patients were diagnosed with osteoporosis in the BPPV (+) group. Meanwhile, 199,257/476,304 patients were diagnosed with osteoporosis in the control group. The results of the pooled analysis showed that the BPPV (+) group had a significantly higher risk of osteoporosis than the BPPV (−) group (OR, 1.73; 95% CI, 1.45–2.06; *p* < 0.01), with high heterogeneity (I^2^ = 94%). A forest plot and further details are shown in Figure 2.

**Figure 3:**
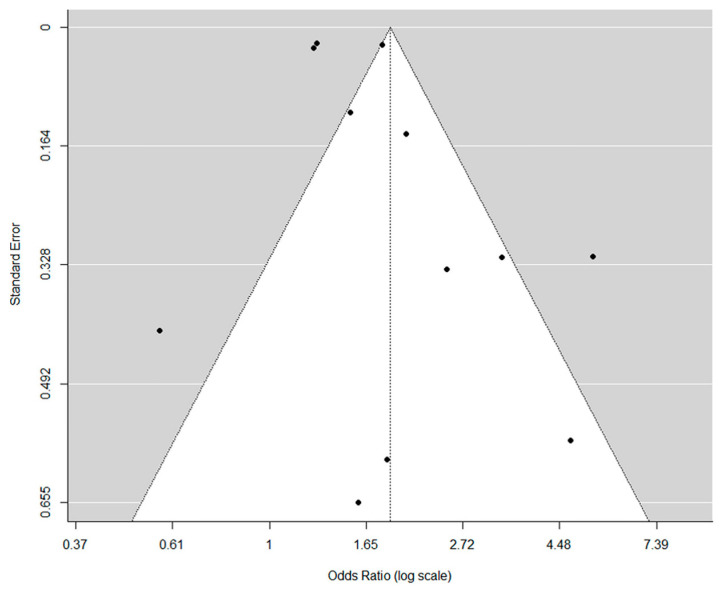
Funnel plot showing the absence of publication bias in the assessment of osteoporosis prevalence in the overall population. The black dots represent a single study included in the analysis.

With regard to the evaluation of publication bias, the results of Egger’s test showed no publication bias upon visual assessment of the funnel plot (Figure 3) with outcomes of osteoporosis prevalence in the overall population (*p* = 0.25; 95% CI, 0.02–0.86).

#### 3.4.2. Subgroup Analyses: Male, Female, and Older Patient (Age ≥ 55 Years) Groups

##### Male Population

**Figure 4:**
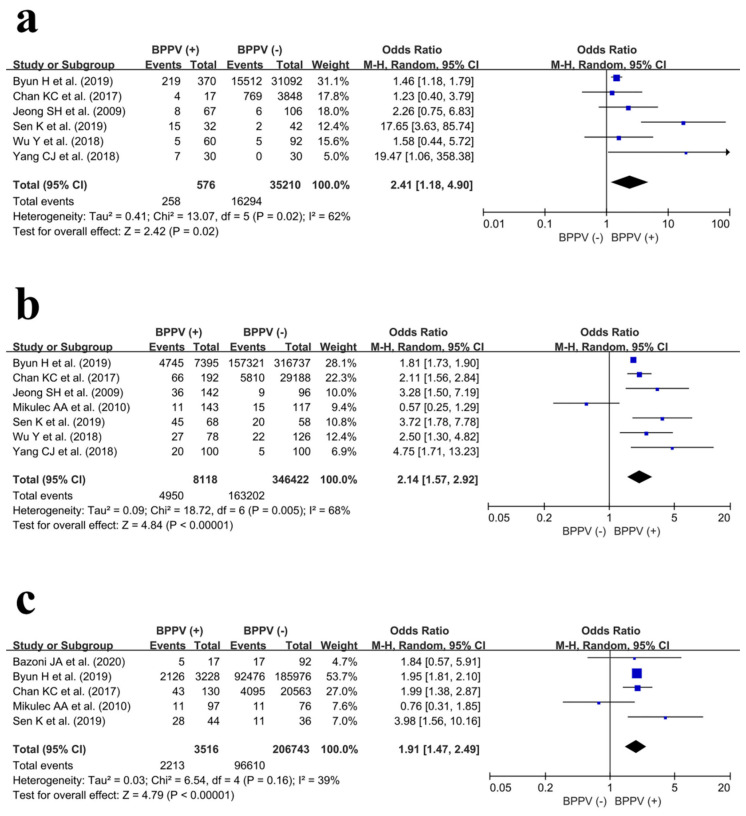
Forest plots showing osteoporosis prevalence between the BPPV (+) and BPPV (−) groups in (**a**) males [4,6,18,22,23,25], (**b**) females [4,6,7,18,22,24,25], and (**c**) older patients (≥55 years) [4,7,17,18,22].

In the male population, data regarding osteoporosis prevalence were extracted from six studies [4,6,18,22,23,25]. Among male participants, 258/576 and 16,294/35,210 patients were diagnosed with osteoporosis in the BPPV (+) group and BPPV (−) group, respectively. The results of the meta-analysis revealed that the BPPV (+) group had a significantly higher risk of osteoporosis compared with the control group (OR, 2.41; 95% CI, 1.18–4.90; *p* = 0.02). Furthermore, the heterogeneity was moderate (I^2^ = 62%). A forest plot and further details are shown in Figure 4a.

##### Female Population

In the female population, data regarding osteoporosis prevalence were extracted from seven studies [4,6,7,18,22,24,25]. Among female patients, 4950/8118 and 163,202/346,422 patients were diagnosed with osteoporosis in the BPPV (+) group and BPPV (−) group, respectively. The results of the meta-analysis revealed that the BPPV (+) group also had a significantly higher risk of osteoporosis compared with the control group (OR, 2.14; 95% CI, 1.57–2.92; *p* < 0.001). Moreover, the heterogeneity was moderate (I^2^ = 68%). A forest plot and further details are shown in Figure 4b.

##### Older Patients (Age ≥ 55 Years)

In the older patient population (age ≥ 55 years), data regarding osteoporosis prevalence were extracted from five studies [4,7,17,18,22]. Among older adults, 2213/3516 and 96,610/206,743 patients were diagnosed with osteoporosis in the BPPV (+) group and BPPV (−) group, respectively. The results of the pooled analysis showed that the BPPV (+) group had a higher risk of osteoporosis compared to the control group (OR, 1.91; 95% CI, 1.47–2.49; *p* < 0.01). Moreover, the heterogeneity was low (I^2^ = 39%). A forest plot and further details are shown in Figure 4c.

## 4. Discussion

The principal finding of the present meta-analysis is that the BPPV (+) group had higher osteoporosis prevalence than the control group. A similar trend was also observed in subgroup analyses as a higher risk of osteoporosis was observed in the BPPV (+) group among male, female, and older patients (≥55 years of age).

BPPV is one of the most commonly recognized vestibular disorders encountered in neurotology clinics and accounts for approximately 20% of all cases of dizziness [27]. The overall prevalence of BPPV in the general population is 1.6% [28]; however, its prevalence is higher among older adults [29]. In terms of pathogenesis, there are two main types of BPPV: Schucknect’s “Cupulolithiasis,” in which the otoliths adhere persistently to the cupula, and Hall’s “Canalolithiasis,” in which the otoliths float in the canal [30]. Although they both explain the mechanisms of BPPV, they do not clarify why BPPV incidence progressively increases in older adults [29,31]. Furthermore, some epidemiologic studies have demonstrated that the prevalence of BPPV is higher in older adults, especially in those between 50 and 60 years of age [32,33]. Osteoporosis prevalence increases with age [34]; moreover, menopause occurs at an average age of 50 years and is associated with a significant increase in bone loss, which increases osteoporosis risk [35]. Interestingly, this also corresponds to the age group with the highest prevalence of BPPV. In a recent study by Juneja et al. [36], osteoporosis was identified as a risk factor for vestibular dysfunction and hearing deficits using vestibular-evoked myogenic potential testing. Theoretically, osteoporosis is a chronic progressive bone disease that leads to reduced mineral density in all bones. Therefore, it can decrease the density of bones, including the temporal bone, cochlear capsule, internal auditory canal, mandible, and bone in the middle ear [36]. Moreover, estrogen deficiency in postmenopausal women leads to further irreversible erosion of bone density [37,38]. BPPV predominantly occurs in female patients and those with calcium metabolism abnormalities, which suggests that there may be a positive relationship between BPPV and osteoporosis. This is also the basis of the present study. Lee et al. [39] conducted a bone turnover marker study and reported that BPPV prevalence in patients with osteoporosis was associated with vitamin D deficiency and high bone turnover rates at the systemic level, which could affect local Ca^2+^ homeostasis in the inner ear. Furthermore, considering that this study is a systematic review and meta-analysis, it was not possible to obtain data. However, taking into account that BPPV risk may be related to Ca^2+^ homeostasis in the inner ear, future extension studies should consider the relevance of vitamin D usage, serum Ca^2+^ levels, and osteoporosis medication in BPPV.

The existence of a likely association between BPPV and osteoporosis has been mentioned previously. In 2014, Yu et al. [10] presented a systematic review of the association between BPPV and osteoporosis. However, as only a few studies had been published, a meta-analysis could not be performed. The authors mentioned that there could be a relationship between BPPV and osteoporosis; however, there was insufficient strong evidence at the time of their study to support this hypothesis. As additional studies have been conducted, we were able to find a significant association between BPPV and osteoporosis in the present meta-analysis.

According to the results of our subgroup analyses, BPPV and osteoporosis showed significant relationships not only in female and older patients aged ≥ 55 years but also in male patients. Although osteoporosis is more prevalent in older women because of hormonal changes after menopause, our meta-analysis showed no difference in BPPV risk between male and female patients with osteoporosis. Interestingly, our study revealed that in addition to the impact of estrogen deficiency on bone quality in BPPV occurrence, there is a close association between bone quality itself and BPPV occurrence in all patients regardless of sex.

The present study has a few limitations. First, we could not perform more detailed subgroup analyses, especially for the T-score of BMD as a continuous variable or for BPPV subtype (e.g., horizontal canal, posterior canal, or anterior canal) because of a lack of study data. Nevertheless, as a meta-analysis is an appropriate method for generating a high level of evidence regarding controversial topics, our synthetic results are meaningful. Second, because of the relatively high heterogeneity of the pooled results, there is a possibility of bias in the interpretation of the meta-analysis results. Third, we could not include randomized controlled trials because of the lack of level I studies. In the future, higher level evidence from prospective randomized trials is required.

Despite these limitations, this meta-analysis supports the positive association between osteoporosis and BPPV. Furthermore, despite numerous studies conducted thus far to investigate the relationship between osteoporosis and BPPV, this study is the first meta-analysis on this topic. The findings of this study can be used in clinical settings to suggest that the presence of BPPV in patients may indicate a potential risk of osteoporosis.

## Figures and Tables

**Table 1:** Study design, demographic data, study details, and MINORS scores.

Author (year)	Study Design	Country	Sample Size (n)		Characteristics of Control Participants in the Control Group	Mean Age	Female Sex	Diagnosis of BPPV	Subtypes of BPPV	Diagnosis of Osteoporosis	T-Score
			BPPV Group	Control Group		Years	%				
Bazoni JA et al. (2020) [17]	PCS	Brazil	17	92	Volunteers of age ≥ 60, both genders	BPPV: 67.0 Control: 68.0	BPPV: 88.2 Control: 58.7	Vertigo complaints, audiological anamnesis, and D–H maneuver	N/A	BMD	N/A
Byun H et al. (2019) [4]	RCS	Republic of Korea	7765	347,829	Matched for age, sex, social status, HTN, and DM	64.5	BPPV: 91.2 Control: 91.2	ICD-10 code	N/A	DXA, qCT, peripheral DXA, and qUS	N/A
Chan KC et al. (2017) [18]	RCS	Taiwan	209	33,036	Matched for age and gender	>20	BPPV: 88.4 Control: 88.4	ICD-9 code	N/A	DXA	N/A
Choi HG et al. (2019) [19]	RCS	Republic of Korea	13,484	53,936	Matched for age, sex, socioeconomic status, HTN, DM, and dyslipidemia	≥50	BPPV: 69.8 Control: 69.8	ICD-10 code	N/A	DXA and qCT	N/A
Jeong SH et al. (2009) [6]	PCS	Republic of Korea	209	202	Volunteers without dizziness and VCF Hx	BPPV: 59.8 Control: 56.3	BPPV: 67.9 Control: 47.5	Vertigo complaints, typical positioning nystagmus, and no other CNS disorders	Horizontal canal, posterior canal, and anterior canal	DXA (LUNAR); T-score ≤ −2.5 from lumbar or femoral areas (WHO definition)	BPPV: −1.7 (female); −1.1 (male). Control: −1.0 (female); −0.7 (male).
Kim SY et al. (2020) [20]	RCS	Republic of Korea	9621	38,484	Matched for age, sex, income, and region of residence	>40	BPPV: 62.6 Control: 62.6	ICD-10 code	N/A	DXA and qCT	N/A
Liao WL et al. (2015) [21]	RCS	Taiwan	644	2160	Matched for age and sex; without vertigo Hx	BPPV: 57.1 Control: 56.7	BPPV: 63.5 Control: 63.5	ICD-9 code	N/A	N/A (ICD-9 code)	N/A
Mikulec AA et al. (2010) [7]	RCS	U.S.	143	117	Patients without BPPV who visited two otology clinics	51–80	100	Clinical evaluation; videonystagmography testing	N/A	N/A	N/A
Sen K et al. (2019) [22]	PCS	India	100	100	Healthy volunteers who want to screen for osteoporosis	BPPV: 48.6 Control: 46.2	BPPV: 68.0 Control: 58.0	D-H maneuver, P-M maneuver, cephalic hyperextension, and audiological assessment	N/A	DXA (T-score ≤ −2.5 from the lumbar or femoral areas)	BPPV: −2.6. Control: −1.8.
Wu Y et al. (2018) [23]	RCS	China	60	92	Matched for age; healthy controls without vertigo/dizziness history	BPPV: 59.4 Control: 62.1	0	Vertigo complaints, D-H maneuver, and supine roll test	N/A	DXA (LUNAR); T-score ≤ −2.5 from lumbar or femoral areas (WHO definition)	BPPV: 1.101 (spine); 0.966 (hip). Control: 1.128 (spine); 1.000 (hip).
Wu Y et al. (2018) [24]	RCS	China	78	126	Matched for age; healthy controls without vertigo/dizziness history	BPPV: 58.4 Control: 58.5	100	Vertigo complaints, D-H maneuver, and supine roll test	Horizontal canal; posterior canal	DXA (LUNAR); T-score ≤ −2.5 from lumbar or femoral areas (WHO definition)	BPPV: −0.11 to −2.42 (spine); −0.04 to −1.73 (hip). Control: 0.70 to −1.45 (spine); 0.36 to 0.96 (hip).
Yang CJ et al. (2018) [25]	RCS	Republic of Korea	130	130	Matched for age and sex; healthy controls without dizziness or fracture Hx	54.9	BPPV: 76.9 Control: 76.9	Vertigo complaints; D-H maneuver	N/A	DXA (LUNAR); T-score ≤ −2.5 from lumbar or femoral areas (WHO definition)	BPPV: −1.6 (female); −0.9 (male). Control: −1.1 (female); −0.7 (male).

Ass, assessment; BMD, bone mineral density; BPPV, benign paroxysmal positional vertigo; DXA, dual-energy X-ray absorptiometry; D–H maneuver, Dix–Hallpike diagnostic maneuver; Hx, history; N/A, nonapplicable; PCS, prospective cohort study; RCS, retrospective cohort study; WHO, World Health Organization.

## Data Availability

Data are available from the corresponding author upon reasonable request.

## Supplementary Materials

The following supporting information can be downloaded at: https://www.mdpi.com/article/10.3390/jpm14030303/s1, Supplement S1. The literature search algorithm and the results from relevant clinical studies.
