# Supplementary material for: Do Patients with Benign Paroxysmal Positional Vertigo Have a Higher Prevalence of Osteoporosis? A Systematic Review and Meta-Analysis
Source: J Pers Med. 2024 Mar 13;14(3):303. doi: 10.3390/jpm14030303 (PMC10971550; doi:10.3390/jpm14030303)
Supplement: Supplementary file 1 [file jpm-14-00303-s001.zip › jpm-2888102-supplementary.pdf]

Supplement S1. The literature search algorithm and the results from relevant clinical studies.

PubMed (17, April, 2023)

	**Search Queries**	**Articles #**
#1	osteoporosis[MeSH Terms]	62,371
#2	osteopenia[Title/Abstract]	10,975
#3	metabolic bone disease[MeSH Terms]	84,883
#4	bone density[MeSH Terms]	60,326
#5	#1 OR #2 OR #3 OR #4	122,651
#6	Benign Paroxysmal Positional Vertigo[MeSH Terms]	1,223
#7	BPPV[Title/Abstract]	1,895
#8	#6 OR #7	2,217
#9	#5 AND #8	50

Embase (17, April, 2023)

	**Search Queries**	**Articles #**
#1	osteoporosis:ti,ab,kw	131,774
#2	osteopenia:ti,ab,kw	19,000
#3	metabolic bone diseas*':ti,ab,kw	4,700
#4	bone density:ti,ab,kw	118,642
#5	#1 OR #2 OR #3 OR #4	213,931
#6	Benign Paroxysmal Positional Vertigo':ti,ab,kw	2,931
#7	BPPV':ti,ab,kw	2,347
#8	#6 OR #7	3,274
#9	#5 AND #8	93

Cochrane Library (17, April, 2023)

	**Search Queries**	**Articles #**
#1	osteoporosis:ti,ab,kw	11,804

#2	osteopenia:ti,ab,kw	1,365
#3	metabolic bone diseas*':ti,ab,kw	1,020
#4	bone density:ti,ab,kw	14,355
#5	#1 OR #2 OR #3 OR #4	20,444
#6	Benign Paroxysmal Positional Vertigo':ti,ab,kw	416
#7	BPPV':ti,ab,kw	291
#8	#6 OR #7	443
#9	#5 AND #8	6
